# From Ischemic Injury to Arrhythmogenic Substrate: Molecular and Histopathological Insights into Post-Infarction Sudden Cardiac Death

**DOI:** 10.3390/life16081299

**Published:** 2026-08-07

**Authors:** Andrea Marzullo, Cecilia Salzillo

**Affiliations:** Pathology Unit, Department of Precision and Regenerative Medicine and Ionian Area, University of Bari “Aldo Moro”, 70124 Bari, Italy; andrea.marzullo@uniba.it

**Keywords:** myocardial infarction, sudden cardiac death, post-infarction remodeling, border zone, cell death, cardiovascular histopathology, arrhythmogenic substrate

## Abstract

Myocardial infarction is a major cause of cardiovascular death and a key substrate for sudden cardiac death. Traditionally, histopathological analysis of infarction has focused on the temporal sequence of morphological changes, from coagulative necrosis to inflammatory infiltrate and cicatricial fibrosis. However, recent molecular studies have highlighted how these processes are tightly regulated by cell death pathways, including apoptosis, autophagy, and ferroptosis, and by electrical and microvascular remodeling mechanisms that contribute to cardiac instability. This review integrates histopathological and molecular evidence relating to post-infarction evolution, with particular attention to the infarct border zone, the privileged substrate for arrhythmogenesis. Key molecular markers and cells involved in the inflammatory response and wound healing are discussed, as well as implications for ventricular reentry circuit formation and sudden cardiac death risk. An integrated understanding of these mechanisms offers innovative perspectives for the identification of predictive biomarkers and the development of therapeutic strategies aimed at reducing post-infarction arrhythmic outcomes.

## 1. Introduction

Myocardial infarction (MI) is a leading cause of morbidity and mortality worldwide and is closely associated with potentially fatal electrical complications. Among these, sudden cardiac death (SCD) is one of the most dramatic manifestations, often representing the first clinical event or occurring during long-term follow-up.

Recent studies in large international cohorts have demonstrated that SCD persists across the spectrum of left ventricular function and is not limited to patients with severe systolic dysfunction [1]. Furthermore, longitudinal studies with prolonged follow-up have highlighted how SCD can occur even decades after infarction, underlining the chronic nature of the post-infarction arrhythmogenic substrate [2].

Pathophysiologically, ischemic heart disease represents the most frequent substrate of SCD, mainly through ventricular arrhythmic mechanisms linked to structural and functional alterations of the myocardium [3]. In this context, the relative role of acute coronary thrombosis versus chronic myocardial fibrosis in the genesis of sudden death remains a matter of debate, suggesting a complex interaction between acute ischemic event and long-term structural remodeling [4].

Pathologically, MI is described as a dynamic process characterized by a temporal sequence of morphological changes, including early ischemic damage, coagulative necrosis, inflammatory infiltrate, and subsequent fibrous scar formation [5,6]. However, more recent evidence indicates that these modifications represent the result of a complex network of molecular events involving mechanisms of cell death, activation of innate immunity and remodeling of the extracellular matrix (ECM) [5,7]. Pathways related to oxidative stress, mitochondrial dysfunction and inflammatory activation contribute significantly to the progression of myocardial damage and the creation of an electrically unstable substrate [8,9,10].

A central role in post-infarction remodeling is played by the so-called infarct border zone, a dynamic interface between injured and viable myocardium that progressively evolves into the principal arrhythmogenic substrate [11]. The structural and electrophysiological characteristics of this region, and their contribution to ventricular arrhythmogenesis and SCD, are discussed in detail in the following sections. In parallel, innovative approaches such as molecular autopsy (MA) have highlighted the importance of genetic and molecular determinants in understanding the mechanisms of sudden death, broadening the role of the pathologist within an integrated morpho-molecular perspective [12,13].

This review aims to integrate histopathological and molecular evidence on post-infarction remodeling, focusing on cell death pathways, inflammatory mediators, fibrosis, microvascular dysfunction, the infarct border zone, and MA. Although individual aspects of post-infarction remodeling, including myocardial fibrosis, infarct border-zone remodeling, ventricular arrhythmogenesis, and SCD, have been extensively reviewed, these processes are often addressed in isolation. Here, we provide a multidisciplinary perspective that combines histopathological evolution, regulated cell death pathways, inflammatory signaling, fibrosis biology, coronary microvascular dysfunction, electrophysiological remodeling, and molecular autopsy into a unified mechanistic framework. Emphasis is placed on the infarct border zone as the pivotal interface where structural, molecular, and electrical remodeling converge to promote ventricular arrhythmogenesis and ultimately SCD.

**Figure 1:**
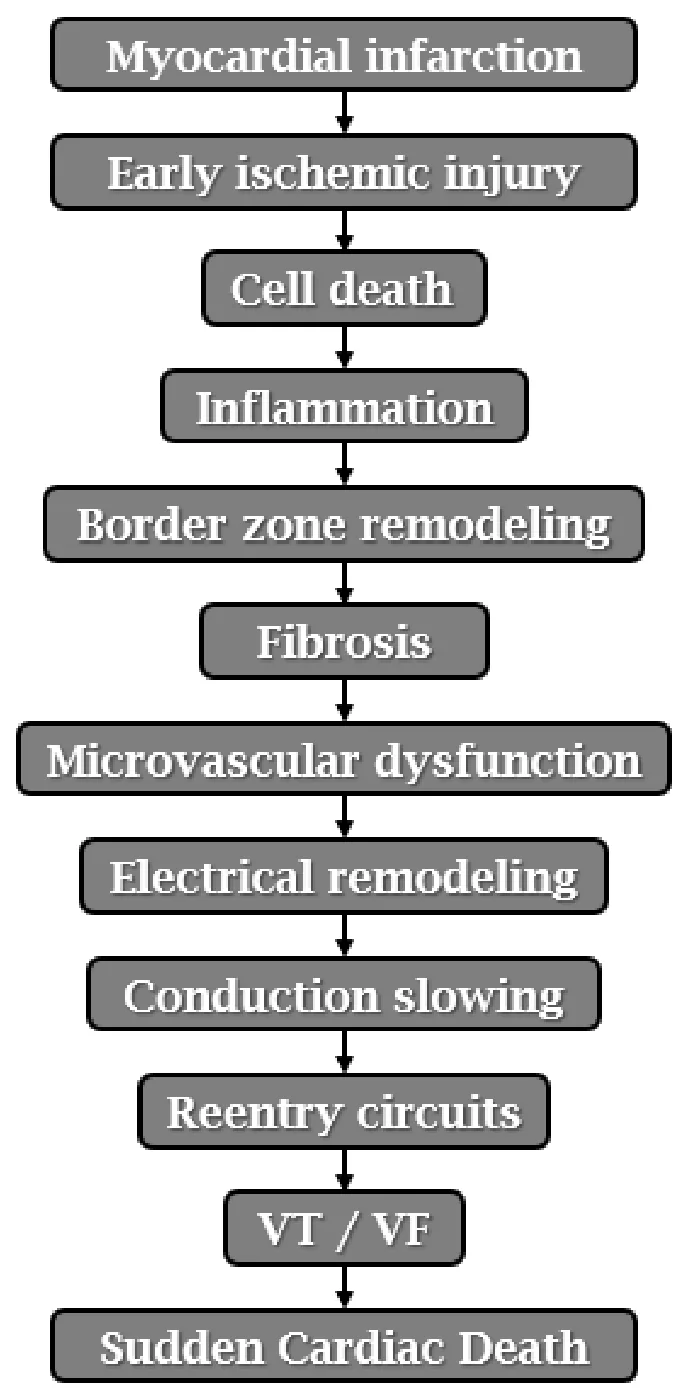
Integrated pathophysiological pathways linking myocardial infarction to ventricular arrhythmias and sudden cardiac death. The figure also illustrates the integrative approach adopted throughout this review, emphasizing the progressive interaction between histopathological, molecular, microvascular, and electrophysiological mechanisms leading to ventricular arrhythmias and sudden cardiac death.

To facilitate the understanding of the complex interactions described in the following sections, Figure 1 provides a schematic overview of the main structural, molecular, and electrophysiological pathways linking myocardial infarction to ventricular arrhythmogenesis and sudden cardiac death. This integrative framework constitutes the principal distinguishing feature of the present review, providing a comprehensive overview of the interconnected mechanisms driving post-infarction arrhythmogenic remodeling.

## 2. Materials and Methods

This narrative review was conducted through a comprehensive literature search using the PubMed/MEDLINE, Scopus, and Web of Science databases. The search focused on studies published primarily between 2021 and 2026, although earlier seminal publications were included when considered essential for understanding the histopathological and molecular mechanisms of post-infarction remodeling. The main search terms included “myocardial infarction”, “sudden cardiac death”, “arrhythmogenic substrate”, “border zone”, “myocardial fibrosis”, “regulated cell death”, “ferroptosis”, “microvascular dysfunction”, “electrical remodeling”, and “molecular autopsy”. Original articles, systematic reviews, meta-analyses, and relevant experimental and clinical studies published in English were considered. The literature was selected based on its scientific relevance, methodological quality, and contribution to the understanding of the mechanisms linking myocardial infarction to ventricular arrhythmogenesis and sudden cardiac death.

## 3. Early Ischemic Injury

In the early stages of MI, minutes to the first hours, histopathological changes are often subtle but represent a crucial moment in the progression of the damage.

The earliest findings are intracellular and interstitial edema, related to increased vascular permeability and cardiomyocyte swelling. Indeed, experimental and clinical studies have demonstrated that myocardial edema appears rapidly after ischemia and can be detected within the first few hours, representing the main tissue manifestation in the initial phases before the inflammatory infiltrate [14].

**Figure 2:**
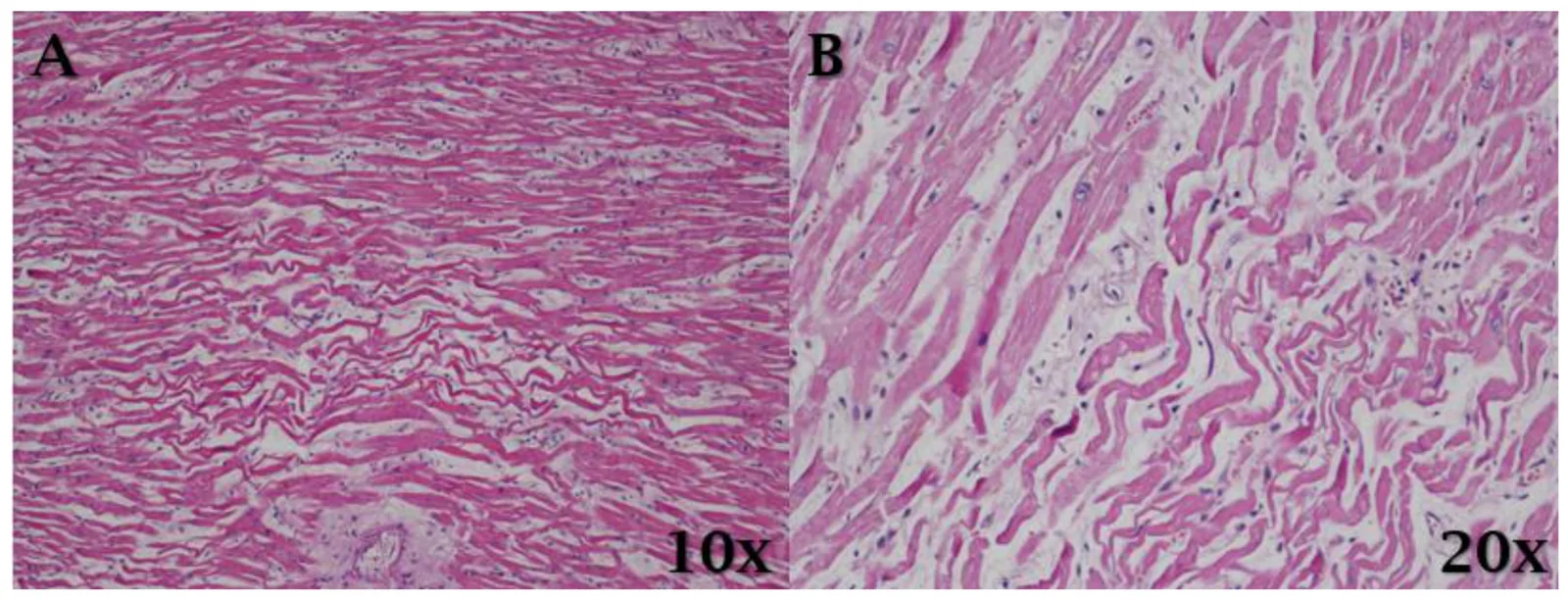
Histological features of acute myocardial infarction. (**A**) Hematoxylin and eosin (H&E) staining, ×10 original magnification, showing the characteristic “wavy fibers” in the ischemic myocardium. (**B**) Higher magnification (H&E, ×20) highlighting the undulating appearance of injured cardiomyocytes, an early morphological hallmark of ischemic injury resulting from traction exerted by the surrounding viable myocardium.

Histologically, the characteristic “waviness” (Figure 2) of the myocardial fibers is also evident, observable within a few minutes of the interruption of coronary flow, probably due to the traction exerted by the surrounding vital myocardium on the ischemic fibers [15]. In parallel, ultrastructural analysis documents early mitochondrial damage, myofibril disorganization and loss of cell membrane integrity, key elements in the transition to irreversible damage [16].

At the molecular level, this phase is dominated by a rapid activation of cellular damage mechanisms mainly related to oxidative stress and mitochondrial dysfunction. The reduction in oxygen supply causes a drastic decrease in ATP production, with accumulation of anaerobic metabolites and alteration of ionic homeostasis [17].

Subsequent reperfusion amplifies the damage through the massive production of reactive oxygen species (ROS), which contributes to lipid peroxidation and cell membrane dysfunction. In this early phase, however, apoptosis is still limited, while reversible alterations and initial necrosis phenomena predominate, suggesting a potential therapeutic window in the first hours after the ischemic event [18].

Furthermore, the early release of DAMPs (damage-associated molecular patterns) from damaged cardiomyocytes contributes to the activation of innate immunity, preparing the ground for the subsequent inflammatory response [17].

Electrophysiologically, these structural and molecular alterations translate into marked myocardial electrical instability. Ischemia rapidly alters electrical conduction through changes in ionic homeostasis and intercellular communication, contributing to membrane depolarization and inhomogeneity of impulse propagation [18].

In parallel, early ischemia induces gap-junction dysfunction, including initial Cx43 remodeling, which contributes to transient electrical instability. The mechanistic implications of Cx43 remodeling during chronic post-infarction remodeling are discussed in Section 5 [19,20].

These changes favor the onset of early ventricular arrhythmias, which represent the main cause of SCD in the acute phase of infarction. Gap junction remodeling and impaired electrical conduction are now recognized as key determinants of arrhythmogenesis in MI [21].

Even minimal structural alterations, such as edema and disorganization of the myocardial tissue, can amplify conduction dispersion and contribute to electrical instability, further increasing the risk of arrhythmia.

The early phase of MI is a critical point in which initially subtle histological alterations reflect profound molecular and electrophysiological changes, setting the stage for the evolution of myocardial damage and the development of potentially fatal arrhythmic complications.

These early electrophysiological disturbances create the first transient arrhythmogenic substrate, facilitating ventricular tachyarrhythmias during the acute phase of MI and contributing to the risk of SCD.

The temporal evolution of histopathological and molecular changes following myocardial infarction is summarized in Table 1.

## 4. Necrosis and Cell Death

**Figure 3:**
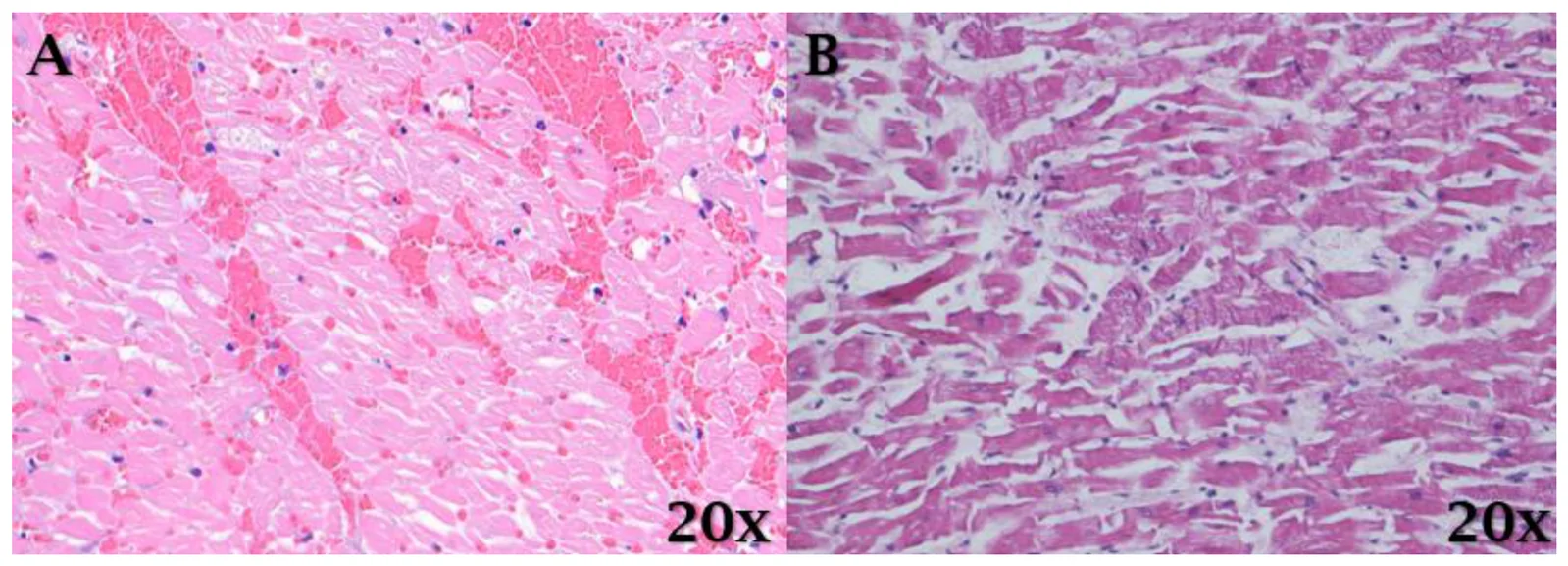
Histological features of acute myocardial infarction. (**A**) H&E staining, ×20 original magnification, showing coagulative necrosis in the acute phase, characterized by cytoplasmic hypereosinophilia, loss of transverse striations, and nuclear disappearance (karyolysis). (**B**) H&E staining, ×20 original magnification, highlighting contraction band necrosis, a characteristic feature observed in the subsequent hours and particularly associated with reperfusion injury and intracellular calcium overload.

In MI, cardiomyocyte loss is a central event and is mediated by a complex interplay of different cell death pathways, including necrosis, apoptosis, autophagy, and ferroptosis. Histopathologically, coagulative necrosis (Figure 3A) is the predominant finding in the acute phases and is characterized by cytoplasmic hypereosinophilia, loss of transverse striations and progressive disappearance of nuclei (karyolysis) preceded by condensation (pyknosis) and fragmentation (karyorrhexis) of nuclear chromatin. In the following hours, contraction band necrosis phenomena (Figure 3B) are observed, particularly associated with reperfusion injury and intracellular calcium overload [14].

At the same time, in the subacute phases, a progressive inflammatory infiltrate is evident, initially neutrophilic and subsequently macrophagic, associated with the removal of cellular debris. At this stage, programmed cell death, particularly apoptosis, becomes more evident and can be documented using immunohistochemical techniques. Autophagy, on the other hand, manifests itself at the ultrastructural level with the presence of autophagic vacuoles and represents an adaptive mechanism that can become maladaptive under conditions of prolonged stress [22].

Recently, ferroptosis has been recognized as a major contributor to ischemic myocardial injury, characterized by mitochondrial ultrastructural alterations, including volume reduction, matrix densification, and loss of mitochondrial cristae, associated with intense lipid peroxidation [23].

In addition to apoptosis and ferroptosis, increasing evidence indicates that other forms of regulated cell death contribute to myocardial injury after infarction. Necroptosis is a programmed necrotic pathway mediated by receptor-interacting protein kinases RIPK1 and RIPK3, leading to MLKL activation, plasma membrane disruption, and amplification of tissue injury [24]. Similarly, pyroptosis is an inflammatory form of programmed cell death triggered by activation of the NLRP3 inflammasome, caspase-1, and gasdermin D, resulting in membrane pore formation and the release of the pro-inflammatory cytokines IL-1β and IL-18. Both pathways enhance post-infarction inflammation, adverse ventricular remodeling, and the development of an arrhythmogenic substrate, representing promising therapeutic targets for limiting myocardial damage and reducing arrhythmic risk [25].

Molecularly, these processes are closely linked to cellular stress mechanisms, including the production of ROS and mitochondrial dysfunction. Oxidative stress represents a central node in the progression of damage, contributing to both necrosis and programmed cell death [18]. Mitochondrial dysfunction, including the opening of the mitochondrial permeability transition pore (mPTP), represents a critical event in the loss of cellular integrity and the transition to irreversible damage [26].

In parallel, cell death, particularly necrotic cell death, is associated with the release of DAMPs, such as HMGB1, extracellular ATP, and mitochondrial DNA. These signals activate innate immunity through receptors such as TLRs and the NLRP3 inflammasome, promoting the production of pro-inflammatory cytokines (e.g., IL-1β, TNF-α) and amplifying the inflammatory response [27].

The implications of these mechanisms are relevant for post-infarction myocardial remodeling. The interaction between cell death and the inflammatory response regulates the removal of necrotic tissue and the subsequent activation of fibroblasts, with the deposition of ECM and scar formation. Histologically, this process evolves towards the replacement of necrotic myocardium with collagen-rich fibrous tissue, which, if disorganized, can contribute to the creation of an electrically unstable substrate and therefore SCD [11].

Beyond tissue loss, cardiomyocyte death promotes electrical discontinuity and heterogeneity of impulse propagation. The resulting structural remodeling favors slow conduction and predisposes to subsequent arrhythmogenic remodeling.

The main cell death pathways involved in myocardial infarction are summarized in Table 2.

## 5. Inflammatory Response

**Figure 4:**
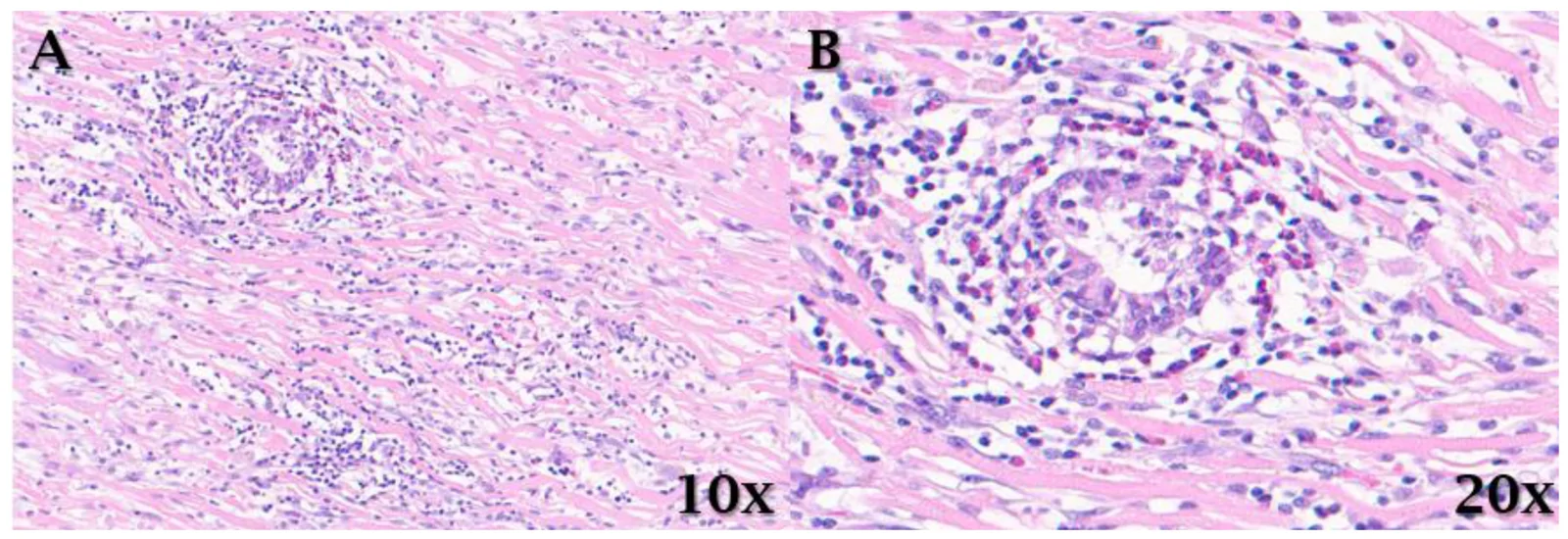
Histological features of inflammatory response following myocardial infarction. H&E staining, ×10 original magnification (**A**) and ×20 original magnification (**B**), demonstrating a mixed inflammatory cell infiltrate with cardiomyocyte necrosis.

The inflammatory response (Figure 4) follows cell death and plays a key role in myocardial remodeling. In the first hours after an ischemic event, a rapid recruitment of neutrophils is observed, which migrate into the necrotic tissue guided by chemokines and damage signals. Neutrophils play an initial role in the removal of cellular debris but, through the release of proteolytic enzymes and reactive oxygen species, can also amplify myocardial damage [28,29].

Subsequently, macrophages become the predominant cell population and show remarkable functional heterogeneity. Traditionally distinguished into M1 (pro-inflammatory) and M2 (pro-reparative) phenotypes, more recent studies have identified additional subtypes, including Mox (a distinct phenotype induced by oxidized phospholipids), Mhem (stimulated by heme and hemoglobin and characterized by a protective and stabilizing effect to injury), and foam-like macrophages, characterized by specific transcriptional and metabolic profiles. This macrophage plasticity is essential for the correct balance between the inflammatory phase and the repair phase [30,31,32].

Molecularly, the inflammatory response is regulated by a complex network of mediators, including cytokines (IL-1β, TNF-α, IL-6), chemokines (CCL2/MCP-1, CXCL8) and inflammasome pathways, particularly the NLRP3 complex, which is activated in response to DAMPs released by damaged cardiomyocytes. Inflammasome activation leads to the maturation and secretion of pro-inflammatory cytokines, amplifying the immune response and contributing to the progression of damage [33,34,35].

These inflammatory processes contribute to subsequent border-zone remodeling, whose structural and electrophysiological consequences are discussed in Section 5 [36,37,38].

The inflammatory response in MI is a dynamic and finely regulated process, in which the interaction between immune cells and molecular mediators determines not only the removal of necrotic tissue but also the evolution of structural and electrical remodeling of the myocardium, with important prognostic implications.

Persistent inflammation also contributes to electrical remodeling through cytokine-mediated ion channel modulation and connexin dysfunction, thereby increasing susceptibility to ventricular tachycardia and ventricular fibrillation.

Key mediators and cellular components of the inflammatory response are reported in Table 3.

## 6. Border Zone of Infarction

The infarct border zone is a critical point in MI, characterized by the coexistence of viable cardiomyocytes, distressed cells, and evolving fibrotic tissue [36]. This heterogeneous architecture generates abrupt transitions between normal myocardium, partially injured cardiomyocytes, and dense fibrotic tissue, creating marked spatial differences in electrical coupling, action potential propagation, and refractoriness. Consequently, the infarct border zone represents the principal structural substrate for post-infarction ventricular arrhythmias [11].

**Figure 5:**
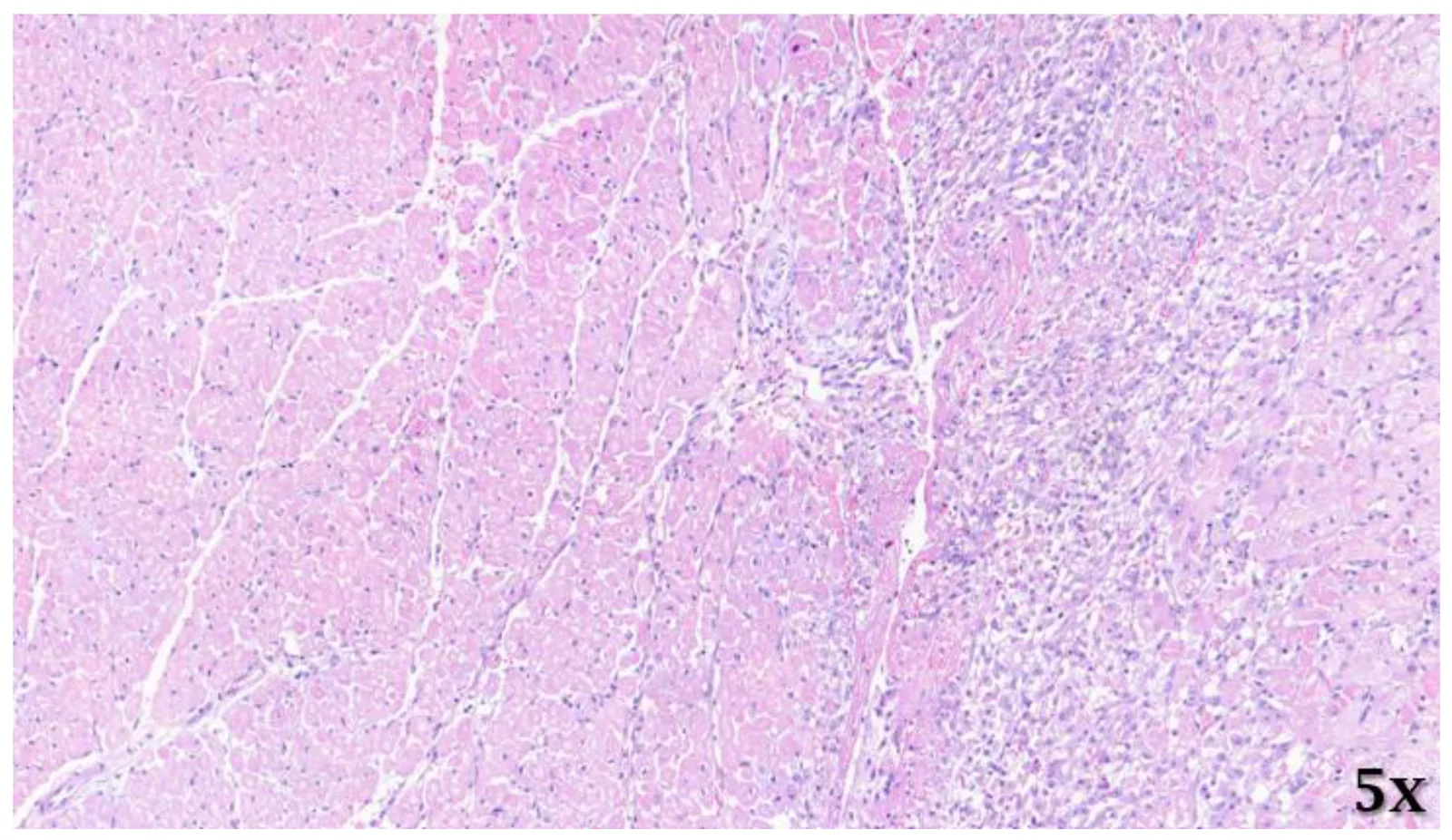
Histological features of the transition zone following myocardial infarction. H&E staining, ×5 original magnification, showing the transition between necrotic and preserved myocardium.

Histopathologically, it represents a transition between necrotic and intact myocardium (Figure 5), with disorganized architecture and variable degrees of replacement fibrosis. This structural heterogeneity is crucial for understanding post-infarction remodeling and its functional consequences [7,37,38].

At the molecular level, the border zone is the site of profound alterations in intercellular and electrical communication. Gap junction remodeling, mainly mediated by connexin 43 (Cx43), represents a key element. Recent studies demonstrate that Cx43 deregulation leads to alterations in electrical conduction and contributes to the development of ventricular arrhythmias through electrical uncoupling between cardiomyocytes and slowing of conduction [39]. Furthermore, changes in Cx43 expression, distribution, and phosphorylation are closely associated with cardiovascular diseases, including ischemic heart disease [40].

In parallel, a significant remodeling of ion channels is also observed in the border zone, with alterations in calcium and potassium fluxes that contribute to electrophysiological inhomogeneity. Experimental studies have highlighted anomalies in calcium dynamics and in the function of gap junctions precisely at the level of the border zone, underlining the role of this region in the genesis of arrhythmias [41]. Furthermore, the interaction between cardiomyocytes and myofibroblasts, through Cx43-mediated coupling, can further impair the propagation of the electrical impulse [42].

These structural and molecular alterations make the border zone the main substrate for the formation of reentry circuits, responsible for post-infarction ventricular arrhythmias. The scar border zone has been shown to be a critical source of ventricular tachycardia, as the combination of slow conduction and tissue heterogeneity favors fragmentation of the activation wave and the perpetuation of arrhythmic circuits [41].

The border zone constitutes a dynamic and highly unstable area, in which the interaction between histopathological alterations and molecular remodeling creates an electrically vulnerable substrate and consequently SCD.

Consequently, the infarct border zone represents the principal anatomical substrate responsible for most post-infarction ventricular tachycardias.

Structural and electrophysiological alterations of the infarct border zone are summarized in Table 4.

### Electrophysiological Basis of Reentry

The transition from structural remodeling to sustained ventricular tachycardia is primarily mediated by electrophysiological alterations occurring within the infarct border zone. Surviving myocardial bundles embedded within fibrotic tissue create narrow conducting pathways characterized by slow and discontinuous impulse propagation. Reduced gap-junction coupling, heterogeneous sodium channel expression, and tissue anisotropy further delay electrical conduction, allowing unidirectional conduction block. These surviving myocardial bundles form protected isthmuses that constitute the critical components of reentrant circuits. In addition, zig-zag conduction and source-sink mismatch promote wavefront fragmentation and electrical instability, facilitating the initiation and maintenance of sustained ventricular tachycardia. These electrophysiological properties also explain the rationale for catheter ablation strategies targeting border-zone conducting channels and protected isthmuses identified by electroanatomical mapping [19,20,21,36,37,38,39,40,41,42].

## 7. Remodeling and Scar Fibrosis

**Figure 6:**
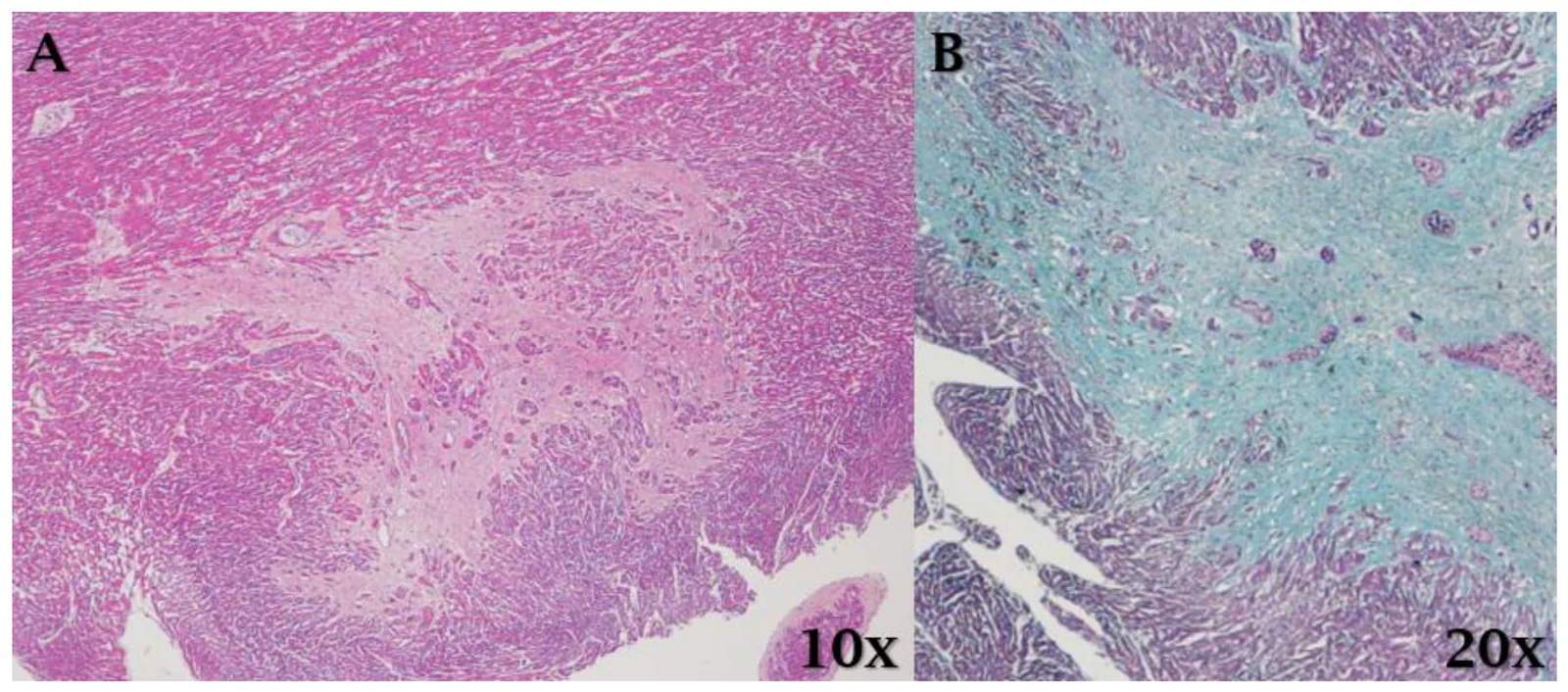
Histological features of post-infarction fibrotic scar formation. (**A**) H&E staining, ×10 original magnification, showing replacement of necrotic myocardium by fibrous scar tissue. (**B**) Masson’s trichrome staining, ×20 original magnification, highlighting collagen deposition within the fibrotic scar.

In MI, post-infarction myocardial remodeling is characterized by a progressive replacement of necrotic tissue with collagen-rich fibrous tissue (Figure 6), a process that is fundamental for structural stability but also potentially arrhythmogenic.

The main effectors of this phase are cardiac fibroblasts, which, once activated and differentiated into myofibroblasts, synthesize ECM components, including type I and type III collagen. Studies highlight how not only the quantity, but also the organization and cross-linking of collagen influence the biomechanical and electrical properties of the myocardium [43,44].

Molecularly, fibrotic remodeling is regulated by highly conserved pathways, including the TGF-β/SMAD system, which represents the main pro-fibrotic axis. Activation of TGF-β promotes the differentiation of fibroblasts into myofibroblasts and stimulates the synthesis of collagen and other matrix proteins [45,46].

Beyond extracellular matrix deposition, accumulating evidence indicates that cardiac fibroblasts exhibit marked phenotypic heterogeneity throughout infarct healing. Activated myofibroblasts are primarily responsible for collagen synthesis and scar formation, whereas inflammatory fibroblasts contribute to immune cell recruitment and modulation of the inflammatory response through cytokine secretion during the early reparative phase. More recently, matrifibrocytes have been identified as a specialized fibroblast population residing within mature scars, where they contribute to long-term extracellular matrix maintenance and scar stabilization. This cellular heterogeneity not only regulates the structural organization of the infarct scar but also influences electrical coupling with adjacent cardiomyocytes, thereby contributing to the development of the post-infarction arrhythmogenic substrate [42,43,44,45,46,47,48,49].

In parallel, the balance between matrix metalloproteinases (MMPs) and their inhibitors (TIMPs) regulates ECM turnover, determining the extent and organization of fibrosis. Alterations in this balance have been associated with maladaptive remodeling and progression to ventricular dysfunction [47,48,49].

Scar fibrosis has significant arrhythmogenic consequences. The disorganized deposition of collagen and the presence of myofibroblasts determine electrical discontinuity and slowing of impulse conduction. The electrophysiological mechanisms by which fibrotic remodeling promotes reentry are discussed in Section 6 [50].

Fibrotic remodeling represents a dynamic and regulated process, in which the interaction between fibroblasts, ECM, and molecular signals determines not only the structural stability of the myocardium but also its electrical vulnerability, with important implications for the genesis of ventricular arrhythmias and SCD.

## 8. Post-Infarction Microvascular Dysfunction

Microvascular dysfunction is a central component of ischemia/reperfusion injury after acute MI.

Histopathologically, it is characterized by capillary rarefaction, edema and endothelial swelling, obstruction of the lumen by platelets and leukocytes, as well as microvascular thrombus formation. These processes determine microcirculatory obstruction and the no-reflow phenomenon, i.e., the lack of tissue reperfusion despite the reopening of the epicardial artery. Furthermore, the presence of intramyocardial hemorrhage and inflammation amplifies microvascular damage and is frequently associated with larger infarcts [51,52,53].

Beyond impairing myocardial perfusion, coronary microvascular dysfunction represents a critical mechanistic link between ischemic injury and arrhythmogenic remodeling. Persistent microvascular obstruction contributes to infarct border-zone expansion, increases scar heterogeneity, and promotes chronic ischemia, thereby facilitating the progressive development of a structurally and electrically unstable substrate.

At the molecular level, hypoxia activates complex adaptive response mechanisms mediated by proangiogenic factors. In particular, the increased expression of VEGF and hypoxia-inducible factors (HIF-1α and HIF-2α) promotes angiogenesis, endothelial permeability and microcirculatory remodeling. However, these responses are often disorganized and insufficient to re-establish adequate perfusion in damaged tissues. In parallel, oxidative stress, endothelial dysfunction and alterations in cellular metabolism contribute to the loss of microvascular integrity [53,54].

The persistence of microvascular dysfunction also contributes to incomplete infarct healing by maintaining areas of residual ischemia within the infarct border zone. Consequently, heterogeneous scar maturation and impaired electrical coupling further enhance conduction abnormalities and increase susceptibility to ventricular arrhythmias.

Clinically, post-infarction microvascular dysfunction is closely related to persistent myocardial ischemia and represents an important negative prognostic determinant. Even in the presence of effective revascularization, microvascular obstruction is associated with increased mortality, adverse ventricular remodeling, and increased risk of major cardiovascular events.

Persistent microvascular obstruction and the no-reflow phenomenon prolong regional ischemia despite successful epicardial reperfusion, promoting infarct border-zone expansion and increasing scar heterogeneity. These alterations amplify structural and electrical remodeling by creating regions of heterogeneous conduction and delayed activation, thereby facilitating ventricular ectopy, ventricular tachycardia, ventricular fibrillation, and ultimately SCD [55,56,57].

## 9. Clinical Implications

The clinical implications of microvascular dysfunction and post-infarction remodeling are now placed in the context of precision medicine, based on the integration of biomarkers and molecular targets to allow for more accurate stratification of arrhythmic risk.

In addition to traditional markers, emerging biomarkers related to endothelial and microvascular dysfunction, such as PCSK9, sST2 and ECM markers, are associated with microvascular obstruction, remodeling and increased risk of arrhythmic events and mortality [58,59,60]. MicroRNAs are emerging as early indicators of fibrosis and post-infarction electrical instability [61]. Biomarkers of hypoxia and angiogenesis, such as HIF-1α and VEGF, have also shown prognostic value in recent studies, reflecting the activation of adaptive pathways but also the severity of ischemic damage [62].

Although several circulating biomarkers have emerged as promising tools for post-infarction risk stratification, their clinical translation remains challenging. Among them, sST2 has demonstrated the strongest clinical evidence owing to its association with adverse ventricular remodeling and heart failure progression and is already incorporated into clinical practice in selected settings. In contrast, biomarkers such as PCSK9, HIF-1α, VEGF, and circulating microRNAs have shown encouraging experimental and observational results, but their diagnostic performance remains questionable. Differences in patient characteristics, timing of biomarker assessment, and analytical methods contribute to inconsistent sensitivity and specificity across studies. Furthermore, large prospective multicenter validation studies are still lacking for most of these biomarkers, limiting their routine use for predicting ventricular arrhythmias and sudden cardiac death. Consequently, their greatest potential currently lies in their integration with imaging findings, electrophysiological parameters, and conventional clinical risk factors within multiparametric precision medicine models, rather than as standalone predictors.

From a therapeutic perspective, the molecular mechanisms involved in post-infarction damage represent increasingly defined targets.

Modulation of inflammation is central, which, through activation of inflammasomes, cytokines (IL-1β, IL-6) and immune signaling, directly contributes to microvascular dysfunction and arrhythmogenic substrate, while their pharmacological control can improve outcomes [52]. Myocardial fibrosis is a key determinant of electrical vulnerability, with ECM biomarkers and pathways such as TGF-β being associated with myocardial stiffness, conduction inhomogeneity, and increased risk of arrhythmias [60,63,64]. Microvascular obstruction itself is closely linked to inflammation and fibrosis and is a powerful predictor of heart failure and mortality [59,65].

An additional therapeutic axis concerns the regulation of cell death and oxidative stress. The processes of necrosis, apoptosis and necroptosis, amplified by redox signals and reactive species, contribute to microvascular damage and the progression of cardiovascular disease. Strategies aimed at limiting these mechanisms or promoting regeneration, for example through HIF-1α/VEGF pathways and pro-angiogenic signals, are emerging as promising approaches to improve perfusion and reduce arrhythmic risk [65,66].

Overall, the most recent evidence indicates that microvascular dysfunction is not only a consequence of ischemic damage, but an active determinant of prognosis, contributing to the persistence of ischemia, structural remodeling and electrical vulnerability, favoring malignant arrhythmias and sudden death. The integration of advanced biomarkers and molecular targets therefore represents a key strategy to personalize treatment and improve clinical outcomes in post-infarction patients.

Therefore, all the molecular pathways discussed in this review converge toward a common arrhythmogenic phenotype, highlighting the importance of integrated risk stratification for SCD.

Emerging biomarkers and molecular targets for post-infarction risk stratification and precision medicine in Table 5.

### Imaging of the Arrhythmogenic Substrate

Cardiac magnetic resonance (CMR) with late gadolinium enhancement (LGE) has become the reference imaging modality for non-invasive characterization of the post-infarction arrhythmogenic substrate. Beyond infarct size assessment, LGE-CMR enables visualization of the peri-infarct gray zone, which consists of surviving cardiomyocytes interspersed with fibrotic tissue and represents the anatomical substrate for slow conduction and reentrant ventricular arrhythmias. Recent studies have demonstrated that the extent of the gray zone and the presence of border-zone conducting channels are independently associated with ventricular tachycardia recurrence and sudden cardiac death. Furthermore, extracellular volume mapping allows quantitative assessment of diffuse myocardial fibrosis, improving arrhythmic risk stratification beyond conventional left ventricular ejection fraction [1,38].

## 10. Sudden Cardiac Death

SCD is a major cause of mortality in patients with MI and continues to pose a significant clinical challenge, despite advances in revascularization and pharmacological therapy.

Recent evidence from large international cohorts indicates that SCD risk persists across the spectrum of left ventricular function and is not adequately predicted by ejection fraction alone, highlighting the limitations of current risk stratification models [66]. Notably, more complex predictive models have not demonstrated a substantial improvement in discriminative ability, suggesting the need for integrated approaches that include molecular data, advanced imaging, and electrophysiological features.

Post-infarction SCD results from the interaction between chronic structural substrate and acute triggers, and a dual model has been proposed in which acute ischemic events (such as coronary thrombosis) and chronic scar remodeling (myocardial fibrosis) contribute synergistically to the genesis of sudden death [4].

The arrhythmogenic substrate underlying post-infarction SCD is primarily determined by the combined effects of structural and electrical remodeling. As discussed in the previous sections, structural and electrical remodeling of the infarct border zone establish the arrhythmogenic substrate underlying post-infarction SCD, whereas acute triggers determine the initiation of malignant ventricular arrhythmias. The interaction between these structural and electrical abnormalities ultimately promotes ventricular fibrillation and increases the risk of sudden cardiac death [67,68].

**Figure 7:**
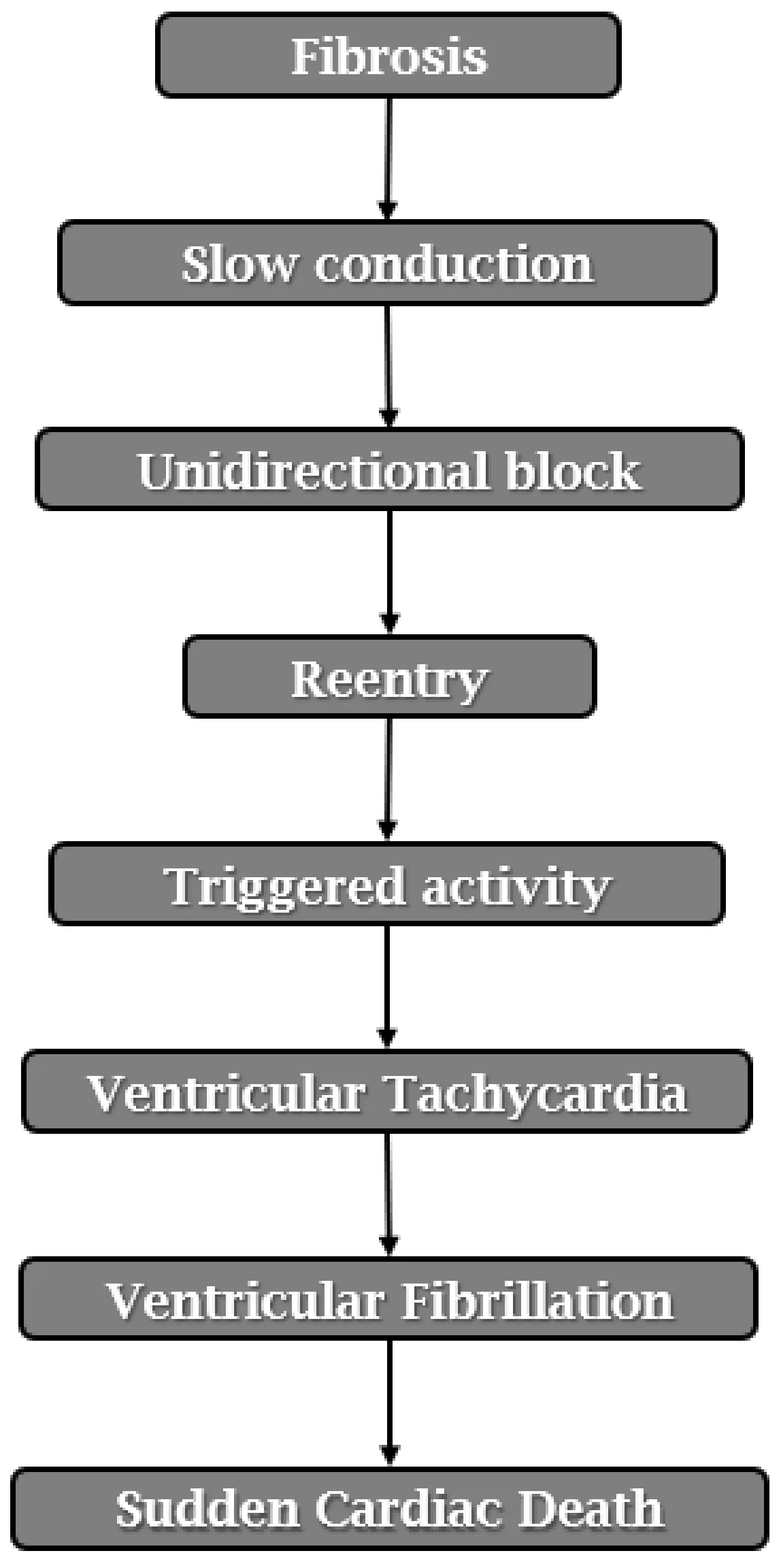
Electrophysiological mechanisms underlying post-infarction ventricular arrhythmogenesis. Myocardial fibrosis and infarct border-zone remodeling promote slow and anisotropic conduction, leading to unidirectional conduction block and the formation of reentrant circuits. Together with triggered activity and electrical remodeling, these mechanisms facilitate ventricular tachycardia, which may degenerate into ventricular fibrillation and ultimately result in sudden cardiac death.

The principal electrophysiological mechanisms linking post-infarction structural remodeling to ventricular arrhythmogenesis are summarized in Figure 7.

In parallel, acute triggers, such as residual ischemia, electrolyte imbalances, and neurohormonal activation, can trigger arrhythmic events on a vulnerable substrate. However, in chronic stages, fibrosis and electrical remodeling become the main risk determinants. As discussed in Section 6, structural remodeling creates slow-conduction channels and protected isthmuses that sustain reentrant ventricular tachycardia [69].

At the cellular level, post-infarction electrical remodeling is characterized by multiple alterations in ion-channel function and intracellular calcium homeostasis. Reduced sodium current (INa) secondary to sodium channel downregulation slows impulse propagation and further contributes to conduction delay within the infarct border zone. In parallel, abnormal calcium handling caused by ryanodine receptor (RyR2) dysfunction and reduced SERCA2a activity promotes spontaneous calcium release from the sarcoplasmic reticulum, generating delayed afterdepolarizations (DADs) and triggered activity. Moreover, action potential prolongation and repolarization heterogeneity favor the development of early afterdepolarizations (EADs), further increasing electrical instability. Together, these alterations enhance repolarization dispersion, facilitate reentrant circuits, and increase susceptibility to sustained ventricular tachycardia, ventricular fibrillation, and SCD.

Molecular and cellular mechanisms also play an important role in modulating arrhythmic vulnerability. Processes such as persistent inflammation, oxidative stress, and cell death contribute to pathological remodeling and progressive electrical instability of the myocardium [70].

Clinically, prevention of SCD is mainly based on the use of implantable cardioverter defibrillators (ICDs) in high-risk patients; however, patient selection remains suboptimal. Recent observational studies confirm that several clinical and structural factors contribute to the risk of sudden death in heart attack survivors, but no single parameter is sufficiently predictive [71]. This has led to the development of more sophisticated strategies that integrate biomarkers, imaging (particularly cardiac magnetic resonance imaging for the quantification of fibrosis), and electrophysiological techniques.

Post-infarction SCD must be considered the result of a multifactorial and dynamic process, in which structural remodeling, molecular alterations and electrical instability interact. A better understanding of these mechanisms, including through the integration of data from clinical and translational studies, represents a fundamental step towards developing more effective and personalized prevention strategies.

## 11. Conclusions and Future Perspectives

MI represents a dynamic and complex process in which histopathological events and molecular mechanisms are closely intertwined, determining the progressive evolution towards an arrhythmogenic substrate responsible for SCD. From the initial phase of ischemic damage, characterized by cellular alterations and electrophysiological dysfunction, to the formation of fibrotic scarring and microvascular remodeling, it clearly emerges how each stage contributes to the creation of a structurally and electrically unstable environment. In particular, the infarct border zone is a key area where tissue heterogeneity and alterations in intercellular communication favor the genesis and maintenance of reentry pathways.

Overall, the evidence reviewed indicates that post-infarction SCD is not the consequence of a single pathological event but rather the outcome of multiple interconnected structural, molecular, and electrical remodeling pathways that progressively establish a highly arrhythmogenic substrate. Rather than focusing on isolated pathological mechanisms, this review provides an integrated framework encompassing structural, molecular, and electrophysiological remodeling involved in post-infarction arrhythmogenesis. This multidisciplinary perspective identifies the infarct border zone as the pivotal mechanistic hub where ischemic injury, regulated cell death, inflammation, fibrosis, microvascular dysfunction, and electrical remodeling converge to promote the development of the arrhythmogenic substrate and ultimately SCD. Despite substantial advances in therapeutic strategies and preventive interventions, arrhythmic risk stratification remains suboptimal, highlighting the need for more personalized and mechanism-based approaches.

Prospects are oriented towards the development of integrated precision medicine models, capable of combining molecular biomarkers, advanced imaging and electrophysiological analyses. The identification of novel predictive biomarkers, including microRNAs, inflammatory mediators, and indicators of extracellular remodeling, may improve the ability to identify high-risk patients early. At the same time, the understanding of molecular mechanisms paves the way for targeted therapies aimed at modulating specific pathogenic pathways, such as inflammation, fibrosis, and oxidative stress.

A further area concerns regenerative and pro-angiogenic strategies, aimed at improving myocardial perfusion and limiting the formation of an arrhythmogenic substrate. In this context, the application of emerging technologies, such as systems biology, artificial intelligence, and MA, could also contribute to a deeper understanding of the determinants of SCD.

In conclusion, a multidisciplinary and integrated approach is key to translating pathogenetic knowledge into effective clinical tools, with the aim of improving the prevention, diagnosis, and treatment of post-infarction arrhythmic complications, significantly reducing the impact of SCD.

## Figures and Tables

**Table 1:** Histopathological and molecular evolution of myocardial infarction.

Time Phase	Histopathological Alterations	Predominant Molecular Mechanisms	Electrophysiological Implications
Minutes-hours	Intracellular and interstitial edema, fiber waviness, early mitochondrial damage.	Oxidative stress, ATP depletion, mitochondrial dysfunction, DAMPs release	Early electrical instability, gap junction alteration (Cx43), acute arrhythmias.
Hours-days	Coagulation necrosis, contraction bands, initial neutrophilic infiltrate.	Necrosis, initiation of apoptosis, activation of innate immunity (TLR, NLRP3), ROS.	Conduction dispersion, increased arrhythmic susceptibility.
Days-weeks	Macrophage infiltrate, debris removal, granulation tissue.	Apoptosis, autophagy, regulated inflammation, fibroblast activation.	Electrical inhomogeneity, ion channel alterations.
Weeks-months	Scar fibrosis, collagen deposition, ECM remodeling.	TGF-β/SMAD activation, MMP/TIMP balance, fibrogenesis.	Slowed conduction, reentry pathways, ventricular arrhythmias.
Chronic	Mature scar, persistent structural disorganization.	Chronic remodeling, residual inflammation, oxidative stress.	Stable arrhythmogenic substrate, risk of sudden cardiac death.

**Table 2:** Major pathways of cell death in myocardial infarction.

Type of Cell Death	Histological Features	Molecular Mechanisms	Pathogenic Role
Necrosis	Hypereosinophilia, nuclear loss, membrane disruption.	ATP depletion, mPTP opening, oxidative stress.	Irreversible damage, DAMP release, inflammation activation.
Apoptosis	Chromatin condensation, nuclear fragmentation.	Caspase activation, mitochondrial pathway.	Controlled cell elimination and contributes to remodeling.
Autophagy	Cytoplasmic autophagic vacuoles.	Lysosome activation, adaptive response to stress.	Protective or harmful if excessive.
Ferroptosis	Mitochondrial alterations (reduction in volume, loss of cristae).	Lipid peroxidation, iron-dependent, ROS.	Emerging role in ischemic and reperfusion injury.
Pyroptosis	Cell swelling, plasma membrane pore formation, membrane rupture, inflammatory cell infiltration, release of intracellular contents.	NLRP3 inflammasome activation, caspase-1 activation, GSDMD pore formation, IL-1β/IL-18 release.	Amplifies inflammation, promotes adverse remodeling and electrical instability.
Necroptosis	Cell swelling, plasma membrane rupture, inflammatory cell infiltration.	RIPK1/RIPK3 activation, MLKL phosphorylation, membrane disruption.	Promotes infarct expansion, fibrosis and adverse ventricular remodeling.

**Table 3:** Mediators of the post-infarction inflammatory response.

	Main Mediators	Function
Immune cells	Neutrophils, macrophages (M1, M2, Mox, Mhem), lymphocytes.	Debris removal, inflammation modulation, and repair.
Cytokines	IL-1β, TNF-α, IL-6	Amplification of the inflammatory response.
Chemokines	CCL2/MCP-1, CXCL8	Leukocyte recruitment.
Inflammasome	NLRP3	IL-1β activation, innate inflammatory response.
DAMPs	HMGB1, extracellular ATP, mitochondrial DNA.	Activation of innate immunity.

**Table 4:** Structural and electrical remodeling in the border zone.

	Alterations	Consequences
Structural	Coexistence of viable myocytes, necrosis, and fibrosis.	Tissue heterogeneity.
Gap junction	Redistribution and phosphorylation of Cx43.	Electrical uncoupling.
Ion channels	Alterations in Ca^2+^ and K^+^ fluxes.	Action potential instability.
Cellular interaction	Myocyte-myofibroblast coupling.	Conduction slowdown.
Functional	Slow and fragmented conduction.	Reentry circuits and arrhythmias.

**Table 5:** Emerging biomarkers and molecular targets for post-infarction risk stratification and precision medicine.

Pathophysiological Process	Representative Biomarkers/Molecular Targets	Clinical Implications	Potential Therapeutic Relevance
Inflammation	IL-1β, IL-6, sST2	Reflect inflammatory activation, adverse ventricular remodeling, and increased arrhythmic risk	Anti-inflammatory therapies targeting cytokine signaling and inflammasomes
Fibrosis and Extracellular Matrix Remodeling	TGF-β, ECM biomarkers	Associated with myocardial fibrosis, conduction heterogeneity, scar maturation, and ventricular arrhythmias	Anti-fibrotic therapies targeting TGF-β signaling and extracellular matrix remodeling
Microvascular Dysfunction	PCSK9, sST2	Correlate with microvascular obstruction, endothelial dysfunction, and adverse clinical outcomes	Strategies aimed at improving microvascular perfusion and endothelial function
Hypoxia and Angiogenesis	HIF-1α, VEGF	Reflect adaptive responses to ischemia and predict infarct healing and myocardial perfusion	Pro-angiogenic and regenerative therapies
Cell Death and Oxidative Stress	Apoptosis-, necroptosis-, ferroptosis-related pathways	Contribute to infarct expansion, oxidative injury, and adverse remodeling	Modulation of regulated cell death pathways and oxidative stress
MicroRNAs	miR-1, miR-21, miR-133	Early biomarkers of fibrosis, electrical remodeling, and ventricular arrhythmias	Precision medicine tools for diagnosis, prognosis, and targeted therapies

## Data Availability

No new data were created or analyzed in this study. Data sharing is not applicable to this article.
